# Effect of Crack Geometry on Tensile Deformation and Local Strain Evolution in X46 Pipeline Steel Thin-Walled Tubes

**DOI:** 10.3390/ma19112265

**Published:** 2026-05-27

**Authors:** Hongqiao Yan, Molin Su, Fangwei Luo, Ruijing Jiang

**Affiliations:** 1 National Center of Technology Innovation for Digita Construction, Huazhong University of Science and Technology, Wuhan 430074, China; 2 School of Civil and Hydraulic Engineering, Huazhong University of Science and Technology, Wuhan 430074, China; 3 CNPC Research Institute of Safety & Environment Technology, Beijing 102206, China

**Keywords:** X46 pipeline steel, thin-walled tube, crack geometry, strain localization, digital image correlation

## Abstract

To investigate the effect of crack geometry on tensile deformation and strain localization in X46 pipeline steel thin-walled tubes, uniaxial tensile tests were conducted on specimens containing prefabricated cracks with different sizes, types, and orientations, and full-field strain evolution was characterized by digital image correlation. The material exhibited a favorable strength-ductility balance, with an average yield strength of 324 MPa, an ultimate tensile strength of 553.5 MPa, and an elongation of 27%. Non-cracked specimens showed three deformation stages: uniform deformation, strain localization, and necking instability. In cracked specimens, strain localization initiated at the crack tips and expanded with increasing displacement. Larger cracks significantly intensified crack-tip strain concentration and enlarged the high-strain zone. Through-wall cracks caused stronger localization and earlier local instability than surface cracks because of the loss of wall continuity, whereas small surface cracks had a limited effect on the final localization path. Crack orientation also affected deformation behavior, and the 45° inclined crack produced the most severe X-shaped localization under combined normal and shear stresses.

## 1. Introduction

Pipeline steels are among the most critical structural materials used in long-distance oil and gas transportation systems, and their in-service performance is directly related to operational safety, environmental risk, and maintenance cost. As transmission pressure continues to increase, service environments become more complex, and pipeline systems move toward higher strength, lighter weight, and longer service life, the identification and control of local damage have become central issues in structural integrity assessment. Compared with uniform strength-based failure, local strain concentration, damage accumulation, and crack growth often determine much earlier whether a structure is approaching a dangerous state. This is particularly true for thin-walled tubular components. In such structures, local deformation is closely coupled with global instability, and the traditional strength parameters alone are no longer sufficient to characterize the actual structural safety margin under service conditions. Among the pipeline steels commonly used in the oil and gas industry, API 5L X46 is a typical low-carbon microalloyed steel. Previous studies have shown that it offers a favorable combination of strength-ductility balance, formability, and weldability, and is therefore widely used in oil and gas transmission pipelines and related pressure-bearing structures [1,2].

In practical engineering, pipeline structures inevitably contain various volumetric or planar defects, among which crack-like flaws are particularly critical and highly evolution-sensitive. They may be introduced during manufacturing, welding, forming, transportation, and installation through inclusions, geometric discontinuities, misalignment, and residual stresses. They may also nucleate and propagate during long-term service under corrosion, cyclic loading, overload, hydrogen-assisted damage, and the combined action of multiple integrity threats [3,4,5,6,7]. Zhou et al. [8] further showed that axial misalignment in welded joints markedly increases stress concentration, reduces fatigue strength, and accelerates fatigue crack growth. Under more complex loading conditions, crack behavior also exhibits pronounced geometric and rate sensitivity. Oikonomidis et al. [9] pointed out that under rapid depressurization in natural gas pipelines, crack propagation and crack arrest are significantly affected by strain-rate-dependent damage. Lou et al. [10] revealed the multiscale evolution of crack-tip plastic zones and overload retardation behavior in X80 pipeline steel under variable-amplitude loading. Ren et al. [11] further demonstrated that Lüders deformation can significantly modify the crack-driving-force response of cracked pipelines. Taken together, these studies indicate that once a crack forms, it no longer acts merely as a passive geometric notch. Instead, it actively alters local load transfer, redirects plastic flow, and changes the path by which the structure approaches instability.

Extensive studies in fracture mechanics and structural integrity assessment have demonstrated the influential role of crack geometry. Ariffin et al. [12] showed that multiple interacting three-dimensional cracks can significantly alter the elastic-plastic fracture response of offshore pipelines under large plastic strain. Jia et al. [13] pointed out that for metallic pipelines subjected to large plastic deformation, even for the same crack size, normalized *J*-integral levels may differ markedly between surface and deeply embedded cracks, indicating the importance of crack location and constraint effects. Zhao et al. [14] established a strain-based *J*-integral formulation for internal circumferential surface cracks in pipelines subjected to internal pressure and large axial deformation, and their subsequent work further showed that crack size and spacing significantly affect the interaction behavior of coplanar double cracks [15]. Zhou et al. [16] quantitatively analyzed the crack-front constraint characteristics of surface cracks in pressurized pipelines. Meanwhile, Paredes et al. [17] described crack initiation and stable crack growth in X70 pipeline steel using a damage-based model. Agbo et al. [18], Najari et al. [19], and Iranmehr et al. [20] further demonstrated, through CT, SENB, and SENT specimens together with XFEM simulations, that fracture toughness, crack extension, and strain-based J-evaluation are all strongly influenced by specimen geometry and constraint conditions. In addition, Zhou et al. [21] proposed an out-of-plane constraint parameter to characterize thickness effects in ductile fracture toughness, whereas Xu et al. [22] showed that stress triaxiality and the Lode parameter can alter the ductile fracture mode of pipeline steel. At the structural scale, Yang et al. [23] demonstrated through full-scale DIC-assisted wide-plate tests that defect configuration directly affects crack-driving force and strain capacity in X80 welded joints. In essence, crack size, crack type, and crack orientation are not secondary descriptors. They are key factors influencing local strain redistribution, localization development, and the degradation of structural reliability.

To experimentally reveal such highly geometry-sensitive local behavior, global load–displacement curves alone are clearly insufficient. Full-field local deformation and strain information must also be obtained. Because it provides non-contact, high-resolution, full-field measurements, digital image correlation (DIC) has become an important tool in crack research and structural integrity assessment. Chen et al. [24] used DIC to quantify the J-integral for through-thickness cracks in circular hollow section joints, showing that DIC can directly support fracture assessment in tubular structures. Chen and Qian [25] subsequently proposed a direct crack identification and sizing approach based on DIC strain fields, indicating that crack location and size can be inferred directly from local strain features. Wang et al. [26] combined DIC and SEM to investigate fatigue crack growth and overload retardation behavior, and showed that crack-tip strain accumulation is directly related to crack propagation. Shahrjerdi and Nazari [27] further demonstrated, through cyclic bending tests on a pressurized 316L pipe tee, that DIC can effectively resolve ratcheting strain accumulation and localized deformation in piping components. At an even finer scale, Leão et al. [28] showed through in situ SEM-DIC testing that local strain concentration can be directly associated with interfacial decohesion and crack initiation in pearlitic steel. Accordingly, DIC provides an effective experimental basis for correlating crack geometry with local strain redistribution and structural integrity degradation.

Despite these advances, existing studies have focused mainly on standard fracture specimens, pressurized pipes, fatigue crack growth, welded-joint defect assessment, or crack-driving-force formulations. Less attention has been paid to the progressive local strain evolution of thin-walled tubular specimens containing prefabricated cracks under monotonic tensile loading. This gap is particularly evident when crack size, crack penetration state, and crack orientation are compared under the same tensile-dominated loading condition. Although the general influence of crack geometry on fracture response is well recognized, the full-field strain-localization process in cracked thin-walled tubular specimens remains insufficiently quantified experimentally.

Accordingly, the novelty of the present work lies not in simply confirming that larger cracks or through-wall cracks are more severe. Instead, this study provides direct DIC-based experimental evidence showing how different crack geometries reconstruct the local strain field and promote the transition from distributed plastic deformation to crack-tip-controlled localization. To this end, X46 pipeline steel thin-walled tubes containing prefabricated cracks with different sizes, types, and orientations were tested under uniaxial tensile loading, and full-field strain evolution was measured using DIC. In addition, quantitative DIC indicators, including the effective maximum first principal strain *ε*_1,*max*_, far-field average strain *ε*_1,*far*_, strain concentration factor *K_ε_*, strain-gradient index *G_ε_*, localization width *ω_loc_*, and normalized high-strain-zone area *η_HS_*, were introduced to numerically compare localization severity among different crack configurations. This approach makes it possible to clarify the distinct roles of crack size, crack type, and crack orientation in influencing crack-tip strain concentration, strain redistribution, localization development, and final instability in X46 thin-walled tubular specimens.

## 2. Experimental Materials and Methods

### 2.1. Experimental Material and Tensile Property Characterization

**Figure 1:**
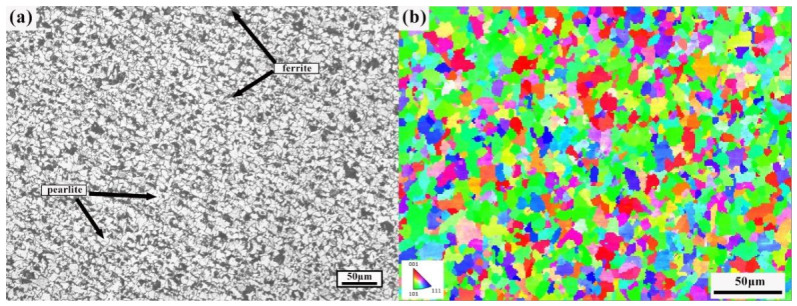
Microstructure and EBSD characterization of X46 pipeline steel: (**a**) optical micrograph; (**b**) IPF map.

API 5L X46 pipeline steel was selected as the experimental material in this study. X46 is a typical low-carbon microalloyed pipeline steel and has been widely used in oil and gas transmission pipelines, pressure-bearing components, and some offshore engineering structures because of its favorable combination of strength-ductility balance, formability, and weldability. Its chemical composition is listed in Table 1. To characterize the initial microstructural state of the material, metallographic specimens were prepared and examined, and a representative micrograph is shown in Figure 1a. The microstructure of the X46 steel consisted mainly of ferrite and pearlite. Ferrite formed the continuous matrix, whereas pearlite was dispersed within the matrix. The overall microstructure was relatively uniform, with no obvious coarse heterogeneous constituents or pronounced banded segregation. The IPF map in Figure 1b provides further information on the crystallographic features. The grains were relatively fine and evenly distributed, with an average grain size of approximately 10 μm. The colors associated with different grain orientations were broadly scattered, and no dominant preferred orientation was observed, indicating that the material exhibited a weak overall texture and a relatively random orientation distribution. Taken together, the X46 pipeline steel shows a fairly uniform ferrite-pearlite microstructure and dispersed crystallographic orientations. Such features are beneficial to achieving favorable overall mechanical properties and a relatively stable deformation response.

**Figure 2:**
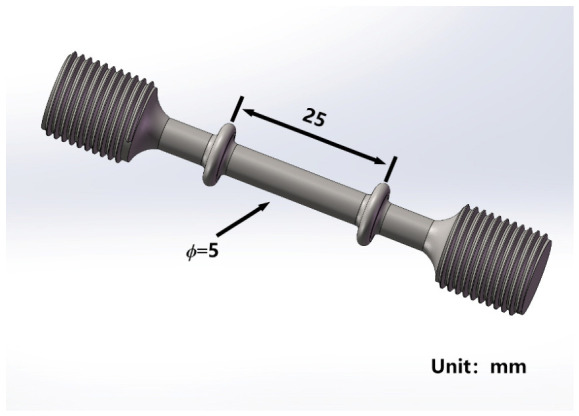
Specimen size for the tensile test.

To determine the basic room-temperature mechanical properties of the X46 base metal and to provide a reference for the subsequent deformation and failure analysis of cracked thin-walled tubular specimens, standard round tensile specimens were first machined for uniaxial tensile testing. The geometry of the tensile specimen is illustrated in Figure 2.

The specimen had a gauge diameter of 5 mm and a gauge length of 25 mm, and threaded ends were adopted to ensure stable loading and to minimize gripping-induced errors as much as possible. Both the specimen design and the room-temperature tensile tests were conducted with reference to ASTM E8/E8M standard [29]. The tests were performed under quasi-static conditions using a DDL-300 electronic (China Machinery Testing Equipment Co., Ltd., Changchun, China) universal testing machine at a loading rate of 0.375 mm/min. Engineering stress–strain curves were obtained accordingly, from which the yield strength, ultimate tensile strength, and elongation after fracture were determined. These parameters are essential. These parameters define the room-temperature mechanical response of the X46 material and provide a baseline for interpreting the tensile deformation behavior and DIC-based full-field strain evolution of cracked thin-walled tubular specimens.

### 2.2. Design and Prefabrication of Cracked Thin-Walled Tubular Specimens

**Figure 3:**
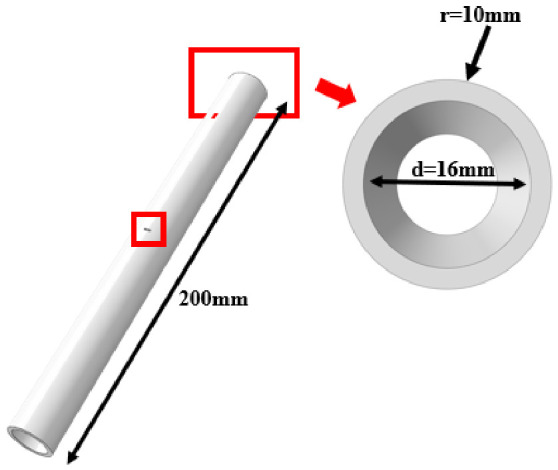
Geometry of the cracked X46 thin-walled tubular tensile specimen.

To systematically investigate the effects of different crack geometrical characteristics on the tensile deformation and strain distribution behavior of thin-walled tubes, X46 pipeline steel tubular specimens were designed based on crack configurations commonly encountered in practical pipeline structures. The specimens were hollow tubes with a total length of 200 mm, an outer diameter of 20 mm, an inner diameter of 16 mm and a wall thickness of 2 mm, as shown in Figure 3. The geometry was straightforward. To ensure stable gripping during axial tension and to prevent local collapse or unintended end deformation, solid steel inserts with a length of 40 mm were fitted into both ends of the tube. This increased the stiffness of the gripping sections and allowed the applied load to be transferred more uniformly to the gauge region. All crack defects were introduced in the middle region along the tube axis, and a symmetric arrangement was adopted as far as possible to reduce the influence of end constraints and boundary effects on the strain-field evolution around the crack.

In this work, the crack geometry was designed from three aspects: crack size, crack orientation, and crack type. Two characteristic crack lengths, 1 mm and 3 mm, were selected to represent defects of different scales and to examine their influence on the strain response. Three crack orientations were considered. These were transverse cracks (0°), longitudinal cracks (90°), and 45° inclined cracks. The transverse crack was perpendicular to the tube axis, the longitudinal crack was parallel to the tube axis, and the 45° inclined crack was introduced to characterize local deformation under combined tensile and shear effects. In addition, two crack types were considered, namely surface cracks on the outer surface and through-wall cracks. By combining these variables, multiple crack configurations were established to systematically compare the local strain concentration and its evolution in thin-walled tubular specimens with different crack geometries. The complete set of crack parameters is summarized in Table 2. For each crack configuration, three parallel specimens were prepared and tested under the same loading and DIC acquisition conditions. The repeated tests showed consistent load–deformation responses and similar strain-localization patterns.

To make the crack-geometry description more general, normalized crack parameters were introduced. The nominal crack length was denoted as *l*, and the normalized crack length was defined as:

(1) $$\lambda =\frac{l}{D}$$

where *D* is the outer diameter of the tube. In the present study, *D* = 20 mm, and the 1 mm and 3 mm cracks correspond to *λ* = 0.05 and *λ* = 0.15, respectively. For the outer-surface cracks, the crack front was idealized as a semicircular profile, and the crack depth ddd was taken as half of the nominal crack length, namely *d* = *l*/2. Therefore, the equivalent weakened area of the outer-surface crack was estimated as:

(2) $$A_{cr,s}=\frac{1}{2}\pi d^{2}$$

For the through-wall crack, the equivalent weakened area was approximated by:

(3) $$A_{cr,t}=lt$$

where *t* is the wall thickness. The remaining ligament ratio was then defined as:

(4) $$R_{lig}=\frac{A_{0}-A_{cr}}{A_{0}}$$

where *A*_0_ = *π*(*D*^2^ − *D_i_*^2^)/4 is the original load-bearing area of the uncracked tube section, *D_i_* is the inner diameter, and *A_cr_* is the equivalent weakened area caused by the crack. For the present tube, *D* = 20 mm, *D_i_* = 16 mm, and *A*_0_ = 113.10 mm^2^. These normalized parameters were used to compare surface and through-wall cracks in terms of their relative crack size and remaining load-bearing ligament.

**Figure 4:**
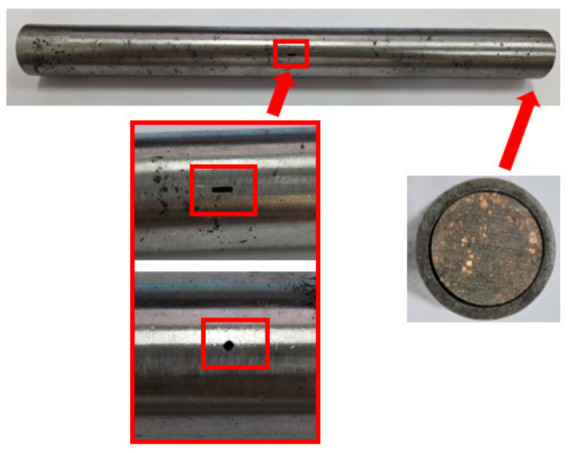
Representative prefabricated crack defects after machining, shown for the 1 mm-45° and 3 mm-90° crack configurations.

The cracked specimens were fabricated by combining wire electrical discharge machining (WEDM) with electrical discharge forming. First, tubular specimens with the required dimensions were cut and machined from the original pipe material by wire cutting. Then, copper electrodes corresponding to the preset crack length, type, and orientation were manufactured and mounted on the electrical discharge forming machine for crack prefabrication. During machining, pulsed voltage was applied between the tool electrode and the workpiece, so that the local material was gradually removed by continuous discharge erosion. A stable discharge gap and feed condition were maintained throughout the process. In this way, crack defects with shapes consistent with the electrode profile were obtained. The process was controllable. The fabricated cracks are shown in Figure 4, where the 1 mm-45° and 3 mm-90° crack configurations are presented as representative examples. It can be seen that the prefabricated cracks had clear contours, accurate locations, and good morphological repeatability.

After crack fabrication, the crack length and crack-tip morphology were examined by optical microscopy to ensure the consistency of the defect geometry among different specimens. The results showed that the dimensional deviation of the cracks was controlled within ±200 μm for all specimens. This level of accuracy met the requirements for specimen repeatability and result comparability in the subsequent tensile tests and DIC-based full-field strain measurements. Overall, the present specimen design and crack prefabrication procedure provided a reliable basis for systematically revealing how crack geometry affects local strain concentration, propagation paths, and failure behavior in thin-walled tubular structures.

### 2.3. Tensile Loading and DIC Strain Measurement

**Figure 5:**
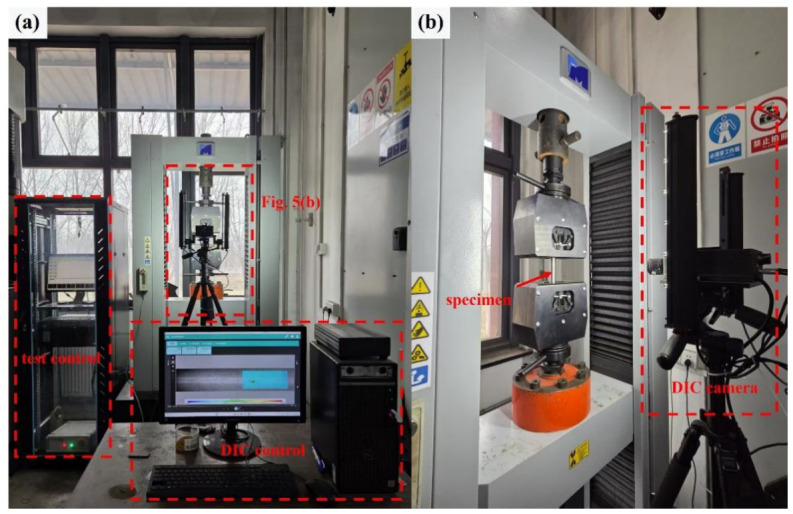
Tensile loading and DIC measurement setup: (**a**) overall system; (**b**) local view of specimen loading and image acquisition.

Uniaxial tensile testing was combined with digital image correlation (DIC) to characterize the global mechanical response of cracked thin-walled tubular specimens under axial tension and to capture local strain evolution in the crack vicinity. The overall testing and measurement setup is shown in Figure 5. As presented in Figure 5a, the setup consisted of a DDL-300 electronic universal testing machine, a test control unit, and a DIC measurement system. Figure 5b shows the local arrangement for specimen loading and image acquisition. The specimen was mounted between the upper and lower grips, and the DIC camera was positioned directly in front of the observation area to continuously record the surface speckle pattern during loading.

The tensile tests were performed on the DDL-300 machine under room temperature, using displacement control at a crosshead speed of 4 mm/min. Special grips were used to secure both ends of the specimen, thereby maintaining stable axial loading and minimizing unintended bending effects. To further examine the possible influence of unintended bending, the strain symmetry of the non-cracked specimen was checked using the DIC strain field. During the early deformation stage, the strain distribution on the two sides of the specimen was approximately symmetric, and no obvious one-sided strain concentration was observed. In addition, the final localization zone of the non-cracked specimen developed near the middle of the gauge section rather than near the grips or along one side of the specimen. These observations suggest that the loading condition was dominated by axial tension and that the influence of additional bending was limited. Load–displacement data were recorded simultaneously to characterize the overall mechanical behavior of the specimen. For full-field strain measurement, a random black-and-white speckle pattern was sprayed onto the outer surface of the observation region before testing. The characteristic speckle size was approximately 50 μm, which satisfied the requirements of DIC analysis in terms of grayscale variation and spatial resolution.

The DIC system consisted of a high-resolution industrial camera, a synchronized acquisition unit, and DIC processing software, with an image acquisition frequency of 0.2 Hz. Before testing, a random black-and-white speckle pattern was sprayed onto the outer surface of the observation region. The characteristic speckle size was approximately 50 μm, which provided sufficient grayscale contrast for image correlation. To ensure comparability among different specimens, the same speckle preparation procedure was used for all tests, including the same surface cleaning procedure, spraying method, and speckle-density control.

During testing, the camera continuously monitored the crack region. The camera mounting position, lens arrangement, and working distance were kept unchanged for all specimens, and the optical axis was maintained approximately normal to the observed surface. This arrangement helped reduce measurement differences caused by changes in imaging geometry. The displacement and strain fields near the crack tip were then obtained by correlating the grayscale information between successive images.

In post-processing, the subset size and step length were selected to balance spatial resolution and strain noise. The same DIC processing parameters were used for all specimens, and principal strain contour maps as well as strain-variation curves along representative paths were obtained. It should be noted that DIC strain measurements near crack tips involve greater uncertainty than those in relatively uniform deformation regions. This is mainly because steep strain gradients, crack opening, local displacement discontinuities, and partial decorrelation may affect the calculated peak strain. Therefore, very high strain values near open cracks were treated as effective DIC strain indicators for comparing localization severity, rather than as exact microscopic material strains at the crack tip. Combined with the synchronized load–displacement data, these measurements were used to analyze the effects of crack size, crack orientation, and crack type on strain distribution and failure behavior.

To obtain a more quantitative comparison of strain localization among different crack configurations, several DIC-derived indicators were extracted from the first principal strain fields. For each cracked specimen, a crack-tip region, *ROI_tip_*, was defined around the two crack tips, and a far-field reference region, *ROI_far_*, was selected away from the crack and the specimen boundaries within the same DIC observation window. For the non-cracked specimen, the central high-strain region was defined as the localization region of interest, and the surrounding relatively uniform deformation region was used as the far-field reference region. All quantitative indicators were extracted from the same DIC strain fields used for the contour plots, so that the visual strain evolution could be compared with numerical measures.

The maximum first principal strain, *ε*_1,*max*_, was used to characterize the severity of local deformation near the crack tip or in the localization region. To evaluate the degree of strain amplification, the strain concentration factor, *K_ε_*, was calculated as follows:

(5) $$K_{\varepsilon }=\frac{\varepsilon_{1,\max}}{\varepsilon_{1,far}}$$

where *ε*_1,*max*_ is the maximum first principal strain in *ROI_tip_* or in the localization region, and *ε*_1,*far*_ is the average first principal strain in *ROI_far_*. A larger *K_ε_* indicates a stronger local strain amplification effect caused by the crack or by necking-induced localization.

To quantify the spatial variation in strain near the crack tip, a strain-gradient index, *G_ε_*, was calculated along representative paths crossing the crack-tip region:

(6) $$G_{\varepsilon }=\frac{\varepsilon_{1,tip}-\varepsilon_{1,far}}{\Delta r}$$

where *ε*_1,*tip*_ is the first principal strain at the crack-tip measurement point, *ε*_1,*far*_ is the first principal strain at a far-field reference point on the same path, and Δ*r* is the distance between these two points. A larger *G_ε_* represents a sharper strain transition from the crack-tip high-strain region to the surrounding lower-strain region, indicating a stronger strain-gradient effect.

The localization width, *ω_loc_*, was determined from the strain profile along the representative path. It was defined as the full width of the region where the first principal strain exceeded half of the maximum strain value:

(7) $$\varepsilon_{1}\geq 0.5\varepsilon_{1,\max}$$

This parameter was used to distinguish broadly distributed deformation from narrow-band localization. A smaller *ω_loc_* indicates that plastic deformation is more strongly confined to a narrow local region.

In addition, the normalized high-strain-zone area, *η_HS_*, was calculated to characterize the spatial extent of the high-strain region:

(8) $$\eta_{HS}=\frac{A_{HS}}{A_{ROI}}$$

where *A_HS_* is the area in which the first principal strain satisfies *ε*_1_ ≥ 0.8*ε*_1,*max*_, and *A_ROI_* is the total analyzed DIC region. The parameter *η_HS_* represents the proportion of the analyzed region occupied by high strain. These indicators, including *ε*_1,*max*_, *K_ε_*, *G_ε_*, *ω_loc_*, and *η_HS_*, were extracted at the same normalized displacement levels whenever possible and were used to quantitatively compare the effects of crack size, crack type, and crack orientation on strain localization.

## 3. Results

### 3.1. Tensile Properties of X46 Steel

**Figure 6:**
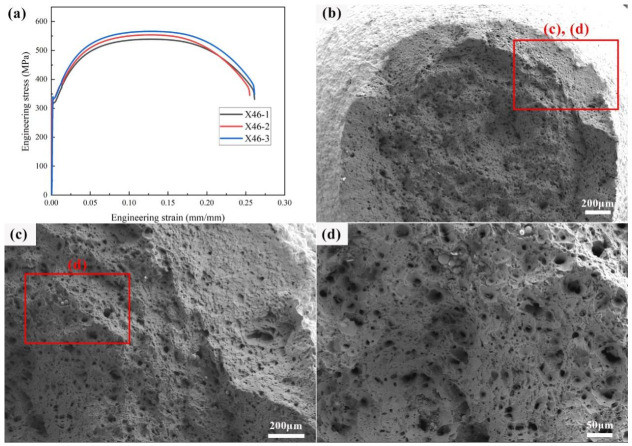
Tensile properties and fracture morphology of X46 steel: (**a**) engineering stress–strain curves; (**b**) macroscopic fracture surface; (**c**,**d**) enlarged fracture features.

Room-temperature tensile tests were first conducted on standard round-bar specimens of X46 pipeline steel to establish the baseline mechanical properties of the material. The corresponding engineering stress–strain curves are presented in Figure 6a, and the detailed mechanical parameters are summarized in Table 3. The three tensile curves follow a highly similar trend, indicating good repeatability of the tests and stable mechanical behavior of the material. All specimens exhibited a typical elastic-plastic response, successively passing through elastic deformation, yielding, strain hardening, and final fracture after necking, and the curves remained smooth without obvious irregular fluctuations.

As listed in Table 3, the yield strengths of specimens X46-1, X46-2, and X46-3 were 308.3, 325.2, and 338.5 MPa, respectively. The corresponding ultimate tensile strengths were 543.5, 554.3, and 562.7 MPa, and the elongations were 27.6%, 26.3%, and 27.1%. The average yield strength, ultimate tensile strength, and elongation were therefore approximately 324.0 MPa, 553.5 MPa, and 27.0%, respectively. Overall, X46 steel exhibits a favorable balance between strength and ductility, allowing it to sustain a relatively high load while maintaining considerable plastic deformation capacity. The material can sustain a relatively high load level while still maintaining considerable plastic deformation capacity, which indicates good deformation capacity and damage tolerance under room-temperature tensile loading. These mechanical properties provide a useful baseline for the subsequent analysis of strain localization behavior in cracked tubular specimens.

Fractographic observations provide further insight into the failure mechanism of the material. As shown in Figure 6b, the macroscopic fracture surface is rough and uneven, indicating evident plastic deformation prior to failure. Higher-magnification views in Figure 6c,d show that the fracture surface is densely covered with dimples of different sizes, accompanied by microvoids, tearing ridges, and traces of local plastic flow. The dimples are numerous and relatively deep. Notably, a small number of spherical inclusions can be observed inside some dimples, suggesting that these inclusions may have acted as nucleation sites for void formation during tensile loading. Interfacial debonding or local stress concentration around such particles likely promoted void initiation and subsequent dimple formation [30]. In contrast, no obvious cleavage facets or river patterns were observed on the fracture surface. This indicates that tensile failure of the X46 steel was not influenced by brittle cracking, but by extensive plastic deformation accompanied by microvoid nucleation and coalescence prior to final rupture.

Taken together, the stress–strain response and fracture morphology indicate that X46 pipeline steel undergoes stable elastic-plastic deformation and fails in a typical ductile manner under room-temperature tension. The dominant fracture mechanism can therefore be identified as ductile failure controlled by microvoid nucleation, growth, and coalescence, with a limited contribution from inclusions that facilitate void nucleation [31]. In other words, the base material itself has good resistance to brittle fracture. This material response is important for interpreting the subsequent tests on cracked thin-walled tubular specimens. The deformation and failure behavior of the cracked specimens should not be regarded as a purely geometric notch effect. Instead, it results from the coupling between crack-induced local load redistribution and the ductile plastic response of the X46 steel. The crack geometry determines where strain concentration is initiated, how the local strain field is reconstructed, and along which path localization develops. By contrast, the material ductility, strain-hardening capacity, and void-growth resistance determine the extent to which the cracked ligament can sustain plastic deformation and redistribute strain before final instability. Accordingly, in the subsequent tensile tests on cracked thin-walled tubular specimens, the observed strain concentration, localization path, and final failure behavior are expected to be influenced primarily by crack geometry and local deformation evolution, rather than by premature brittle failure of the matrix material.

### 3.2. Strain Distribution of the Non-Cracked Specimen

**Figure 7:**
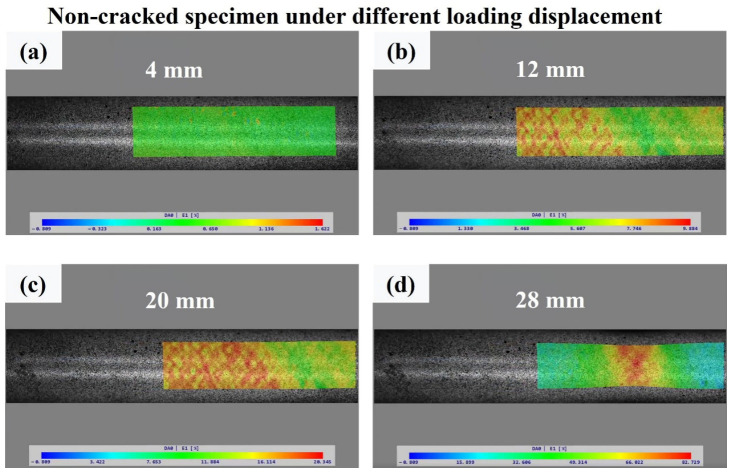
Surface strain distribution of the non-cracked specimen at different loading displacements: (**a**) 4 mm; (**b**) 12 mm; (**c**) 20 mm; (**d**) 28 mm.

To clarify the intrinsic deformation behavior of the X46 thin-walled tubular specimen under uniaxial tension in the absence of prefabricated cracks, the surface strain distributions of the non-cracked specimen at different loading displacements were analyzed, as shown in Figure 7 and Table 4. These results also provide a baseline for evaluating the disturbance introduced by crack geometry in the cracked specimens. Overall, the strain field exhibits a clear stage-wise evolution during tensile loading. Deformation is relatively uniform at the early stage, then gradually becomes nonuniform with increasing displacement, and finally develops into a concentrated localization core near the middle of the gauge section. This indicates that the plastic deformation of the non-cracked specimen does not remain spatially uniform throughout the entire loading process, but progressively evolves from globally coordinated deformation to locally dominated deformation.

At a loading displacement of 4 mm, as shown in Figure 7a, the overall strain level on the specimen surface remains low, and the strain field in the observation region is relatively uniform, with only slight local fluctuations. Quantitatively, the effective maximum first principal strain is approximately 0.715%, the strain concentration factor is 1.64, and the strain-gradient index remains as low as 0.0026%/mm. These results indicate that the specimen is still in a stable stage of nearly uniform deformation, in which the applied load is mainly accommodated by coordinated plastic deformation of the gauge section and no pronounced local strain concentration has yet developed. When the displacement increases to 12 mm, Figure 7b shows a noticeable increase in the strain level, and the strain distribution begins to transition from a uniform to a nonuniform state. A relatively high-strain region starts to emerge near the middle of the gauge section and gradually spreads into the surrounding area. The maximum first principal strain increases to approximately 9.884%, while the localization width remains approximately 56.3 mm. This suggests that the specimen has entered a stage of plastic strain development and redistribution, but the deformation is still distributed over a relatively broad region rather than being confined to a narrow localization band.

When the loading displacement is further increased to 20 mm, the strain heterogeneity becomes much more pronounced, as shown in Figure 7c. The maximum first principal strain further increases to approximately 20.345%, and the high-strain region in the middle section becomes increasingly distinct. The high-strain-zone area fraction also increases to 50.26%, indicating that a large portion of the analyzed region has entered a high-strain state. However, the localization width remains approximately 52.4 mm, suggesting that plastic deformation has not yet evolved into severe narrow-band localization. Instead, the strain field is characterized by broad plastic strain redistribution, although the middle region has already become the dominant deformation zone. This indicates that the deformation control mechanism of the non-cracked specimen is gradually shifting from globally coordinated deformation to locally dominated deformation. After further loading to 28 mm, the deformation becomes markedly localized, as shown in Figure 7d. The high-strain region becomes more intense and more concentrated near the center of the gauge section, forming a pronounced localization core. Quantitatively, the maximum first principal strain increases sharply to approximately 74.053%, the strain concentration factor increases to 2.90, and the strain-gradient index rises to 2.89%/mm. Meanwhile, the localization width decreases to approximately 26.8 mm. These changes indicate that the deformation mode has shifted from broad plastic strain redistribution to severe localized necking in the central gauge region. At this stage, further plastic deformation is mainly confined to the localized necking zone, while strain accumulation in the surrounding regions becomes relatively limited. Such a strain-field pattern is characteristic of the stage immediately preceding necking instability, indicating that the specimen has entered a regime of severe localized deformation [32].

Overall, the strain evolution shown in Figure 7 and the quantitative DIC indicators in Table 4 indicate that the non-cracked X46 thin-walled tubular specimen undergoes three successive stages during tensile loading: uniform deformation, strain redistribution, and enhanced localization. The final high-strain zone consistently develops near the middle of the gauge section rather than near the ends or observation boundaries. This not only suggests that the loading condition is well aligned and that the influence of end constraint or additional bending is limited, but also indicates that, in the absence of crack-induced disturbance, the instability position is influenced primarily by the intrinsic plastic evolution of the material together with geometric instability. Therefore, if premature localization, high-strain-zone deflection, or changes in localization path are observed in the cracked specimens, such phenomena can be more directly associated with the disturbance, reconstruction, and amplification of the local strain field caused by crack defects.

### 3.3. Effect of Crack Size on Strain Distribution

**Figure 8:**
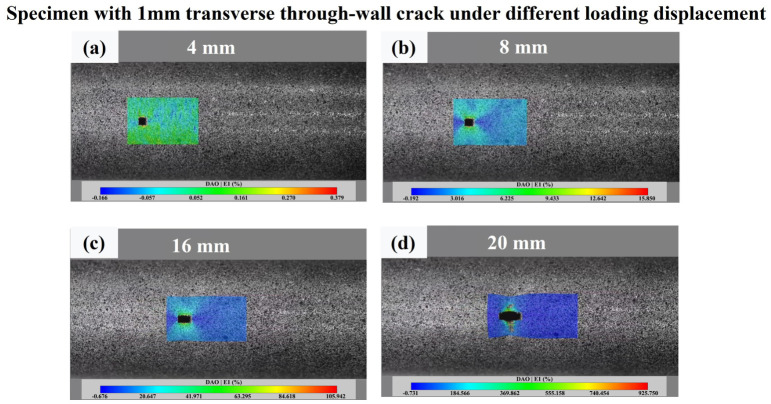
Surface strain distribution of the specimen with a 1 mm transverse through-wall crack at different loading displacements: (**a**) 4 mm; (**b**) 8 mm; (**c**) 16 mm; (**d**) 20 mm.

**Figure 9:**
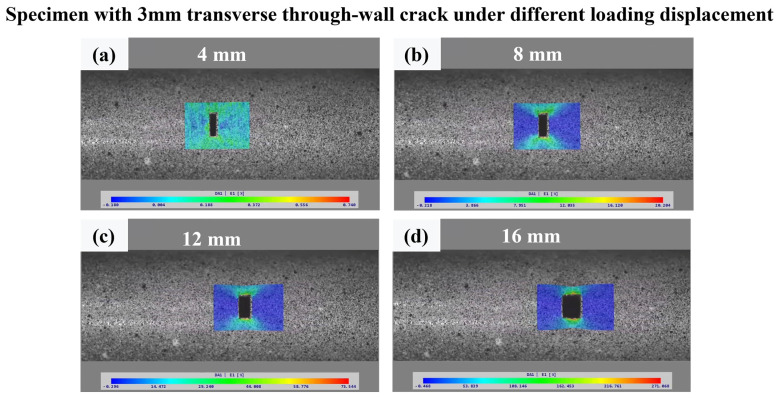
Surface strain distribution of the specimen with a 3 mm transverse crack at different loading displacements: (**a**) 4 mm; (**b**) 8 mm; (**c**) 12 mm; (**d**) 16 mm.

To evaluate the effect of through-wall crack size on local strain distribution and its evolution in thin-walled tubular specimens, specimens containing 1 mm and 3 mm through-wall transverse cracks were compared, as shown in Figure 8 and Figure 9 and Table 5 and Table 6. The strain maps in these figures are local enlargements of the crack vicinity, and the observed regions are still located in the middle of the gauge section. Therefore, compared with the non-cracked specimen, the main difference is not a shift in the macroscopic location of localization, but a change in the way strain becomes concentrated within the same middle gauge region. In the non-cracked specimen, strain gradually accumulates in the middle of the gauge section and eventually forms a localization core. In the specimens containing through-wall transverse cracks of different sizes, however, strain within the same region concentrates preferentially at the crack tips, giving rise to a crack-tip-controlled localization pattern.

For the specimen containing a 1 mm transverse through-wall crack, Figure 8 shows a continuous evolution from mild local perturbation to clear localization. At a displacement of 4 mm, a slight local increase in strain can already be identified near the crack tips, although the overall strain field remains relatively continuous. This indicates that the crack has started to affect local deformation, but has not yet fully dominated the plastic flow. When the displacement increases to 8 mm, the high-strain regions near both crack ends become more distinct, and strain begins to accumulate preferentially in the crack-tip regions, showing that the crack increasingly influences the strain distribution within the middle part of the gauge section. At 16 mm, these high-strain regions expand further and the strain field becomes markedly more heterogeneous. Local deformation no longer develops in a broadly distributed manner, but increasingly evolves around the crack tips. At 20 mm, the high-strain zones become strongly concentrated at both crack ends, and the crack vicinity turns into the principal region accommodating subsequent plastic deformation, indicating that the specimen is approaching local instability. Thus, although the 1 mm crack does not alter the fact that localization still develops in the middle gauge section, it clearly changes the spatial organization of strain within that region.

To further quantify the strain evolution shown in Figure 8, several DIC-derived indicators were extracted from the first principal strain field, as summarized in Table 5. For the 1 mm transverse through-wall crack, the strain concentration factor *K_ε_* remains higher than 2.4 throughout the loading process, indicating that the crack tip continuously acts as a local strain-amplification site. At 4 mm, although the effective maximum first principal strain is still low, approximately 0.34%, *K_ε_* has already reached about 2.83, suggesting that the through-wall crack begins to perturb the local strain field at the early loading stage. With increasing displacement, the strain-gradient index *G_ε_* increases from approximately 0.04%/mm at 4 mm to 5.7%/mm at 16 mm, showing that the transition from the crack-tip high-strain region to the surrounding lower-strain region becomes progressively sharper. At 20 mm, *K_ε_* further increases to approximately 3.52, while the localization width decreases to about 5.0 mm, confirming that plastic deformation is increasingly confined to the crack-tip region.

It should be noted that the very high effective peak strain at 20 mm does not necessarily represent the true microscopic material strain at the crack tip. At large loading displacement, the crack opens significantly and local displacement discontinuity develops across the crack flanks. Because DIC calculates strain from spatial displacement gradients within a finite subset, part of the crack-opening displacement and local decorrelation near the crack edge may be interpreted as an apparent strain concentration. Therefore, the peak value at the final loading stage should be regarded as an effective DIC strain indicator for comparing localization severity, rather than as the absolute physical strain of the material at the mathematical crack tip.

A stronger size effect is observed for the specimen containing a 3 mm transverse through-wall crack, as shown in Figure 9. Even at a displacement of 4 mm, relatively distinct high-strain zones have already formed at both crack tips, indicating that the larger crack imposes a much stronger local disturbance on the strain field in the middle gauge section from the early stage of loading. At 8 mm, these high-strain zones intensify rapidly and develop a more pronounced symmetric pattern at the two crack ends, reflecting faster plastic-zone development and more severe strain redistribution near the crack tips. By 12 mm, the high-strain regions in the crack-tip vicinity become further intensified and exert stronger control over the overall morphology of the strain field, indicating a clear departure from the more continuous strain evolution observed in the non-cracked specimen. At 16 mm, strain becomes even more concentrated at both crack ends and a distinct localization band is established, indicating that the specimen enters a severe local deformation stage at an earlier loading displacement. Compared with the 1 mm through-wall transverse crack specimen, the 3 mm through-wall transverse crack produces a more pronounced crack-tip-controlled localization mode and earlier signs of instability.

The quantitative indicators extracted from Figure 9 further demonstrate the stronger localization effect caused by the 3 mm through-wall crack, as summarized in Table 6. At the same displacement of 4 mm, the 3 mm crack shows a higher effective maximum first principal strain, approximately 0.55%, than the 1 mm crack, indicating a stronger early-stage perturbation of the local strain field. When the displacement increases to 8 mm, the difference becomes more pronounced: the 3 mm crack exhibits an effective maximum first principal strain of approximately 16.1% and a strain concentration factor of approximately 4.16, both higher than those of the 1 mm crack at the same displacement. The strain-gradient index also increases to approximately 1.53%/mm, reflecting a sharper strain transition around the crack tips. At 12 mm and 16 mm, the localization width decreases to approximately 4.8 mm and 4.5 mm, respectively, indicating that plastic deformation becomes increasingly confined to a narrow region near the crack tips. These results confirm that the 3 mm through-wall crack induces earlier and stronger crack-tip-controlled localization than the 1 mm through-wall crack.

Taken together, Figure 8 and Figure 9, together with the quantitative indicators in Table 5 and Table 6, show that increasing the through-wall crack size does not change the general localization position in the middle gauge region. However, it substantially intensifies strain concentration and accelerates the development of crack-tip-controlled localization. This size effect can also be interpreted using the normalized crack length *λ* = *l*/*D* and the remaining ligament ratio *R_lig_* = (*A*_0_ − *A_cr_*)/*A*_0_, where *l* is the nominal crack length, *D* is the tube outer diameter, *A*_0_ is the original load-bearing area of the uncracked tube section, and *A_cr_* is the equivalent weakened area caused by the crack. For the present specimens, *D* = 20 mm; therefore, the 1 mm and 3 mm through-wall cracks correspond to *λ* = 0.05 and *λ* = 0.15, respectively. Because the effective weakened area of a through-wall crack can be approximated as *A_cr_* = *lt*, where *t* = 2 mm is the wall thickness, the remaining ligament ratio decreases from approximately 0.982 for the 1 mm crack to approximately 0.947 for the 3 mm crack. The 1 mm crack behaves mainly as a local perturbation, and its influence increases gradually with loading. In contrast, the 3 mm crack reorganizes the strain field at an earlier stage and rapidly turns the crack tips into the dominant zones of plastic deformation. This is mainly because a larger through-wall crack reduces the effective local load-bearing section and increases the stress concentration near the crack tips [11,33]. In normalized terms, the increase in *λ* and the decrease in *R_lig_* indicate that the 3 mm crack removes a larger fraction of the effective load-bearing ligament and produces a stronger local load-transfer disturbance. As a result, plastic strain accumulates more readily at both crack ends and evolves more quickly into a stable localization band. Quantitatively, the 3 mm crack generally exhibits higher *ε*_1,*max*_, larger *K_ε_*, steeper *G_ε_*, and smaller *ω_loc_* than the 1 mm crack at comparable loading stages. For example, at 8 mm, *K_ε_* increases from approximately 2.42 for the 1 mm crack to approximately 4.16 for the 3 mm crack. Meanwhile, the localization width of the 3 mm crack decreases progressively from about 6.0 mm to 4.5 mm during loading, indicating increasing confinement of plastic deformation near the crack tips. These results demonstrate that the crack-size effect is not only visible in the strain-contour morphology, but also quantitatively reflected by the DIC-derived localization indicators. The normalized crack-length and ligament-ratio parameters further show that this effect is not only related to the absolute crack length, but also to the relative crack size and the remaining load-bearing capacity of the thin-walled tube section. Therefore, through-wall crack size can be regarded as a key geometrical factor influencing strain-field evolution and failure sensitivity in cracked thin-walled tubular specimens.

### 3.4. Effect of Crack Type on Strain Distribution

To evaluate the effect of crack type on local strain distribution and its evolution in thin-walled tubular specimens, the transverse outer-surface crack specimens analyzed in this section were compared with the corresponding transverse through-wall crack specimens discussed in Section 3.3. The results show that crack type has a pronounced influence on strain-field evolution. The difference is reflected not only in the intensity of strain concentration, but also in the onset of localization, its spatial extent, and the degree to which the crack vicinity controls the overall deformation mode. In general, outer-surface cracks also induce local strain concentration near the crack tips, but their effect is clearly weaker than that of through-wall cracks. This is especially true for small surface cracks, whose influence may be insufficient to dominate the final localization path throughout the entire loading process.

**Figure 10:**
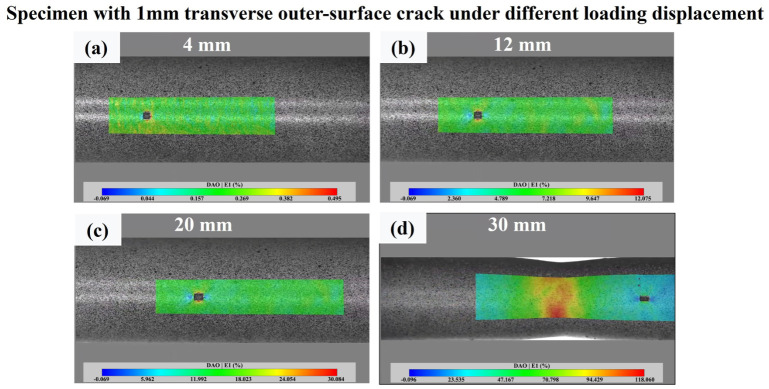
Surface strain distribution of the specimen with 1 mm transverse outer-surface crack at different loading displacements: (**a**) 4 mm; (**b**) 12 mm; (**c**) 20 mm; (**d**) 30 mm.

For the 1 mm transverse outer-surface crack specimen, the strain evolution in Figure 10 and Table 7 differs substantially from that of the 1 mm through-wall transverse crack specimen. At a loading displacement of 4 mm, only a weak local perturbation is observed around the crack, and the overall strain field remains relatively uniform, indicating that the outer-surface crack has only a limited influence on local deformation at this stage. This interpretation is supported by the quantitative DIC indicators: the effective maximum first principal strain is only approximately 0.22%, while the strain concentration factor *K_ε_* is approximately 1.47 and the strain-gradient index *G_ε_* is only 0.015%/mm. These values indicate that the 1 mm outer-surface crack produces only weak early-stage strain amplification because the remaining wall-thickness ligament can still sustain and redistribute the applied load. When the displacement increases to 12 mm, a more visible high-strain region develops near the crack vicinity, but it remains confined and does not establish the strong and stable crack-tip-controlled zones observed in the through-wall crack specimen. At this stage, *ε*_1,*max*_ increases to approximately 6.5%, but *K_ε_* remains low at about 1.38, suggesting that the increase in strain is still mainly associated with distributed plastic deformation rather than severe crack-tip localization. At 20 mm, strain concentration near the crack becomes more evident, yet the high-strain region is still largely restricted to the immediate neighborhood of the crack and does not substantially restructure the strain field across the central gauge region. The corresponding *K_ε_* remains approximately 1.35, and the localization width is still about 41.5 mm, further confirming that the strain field has not yet evolved into a narrow crack-tip-controlled localization band. A more revealing feature appears at 30 mm, where a broader high-strain band develops in the middle of the specimen and gradually becomes the dominant localization region, whereas the small outer-surface crack does not remain the dominant high-strain site throughout deformation. Although the effective maximum first principal strain increases markedly to approximately 82.0% and *K_ε_* rises to approximately 2.38 at this stage, the high-strain zone is mainly associated with global necking in the middle gauge region rather than direct crack-tip-controlled fracture. Therefore, the high strain measured at 30 mm should be interpreted as an effective DIC indicator of final necking localization, not as evidence that the 1 mm surface crack has become the primary failure-control defect. This indicates that failure is still influenced mainly by necking rather than by direct fracture at the crack location. The 1 mm surface crack is too small to generate a sufficiently strong notch effect. Moreover, because it does not penetrate the wall thickness, the remaining internal ligament can still carry and redistribute the applied load. As a result, crack-tip localization is weakened, while global plastic flow and geometric necking instability continue to dominate the final failure process.

**Figure 11:**
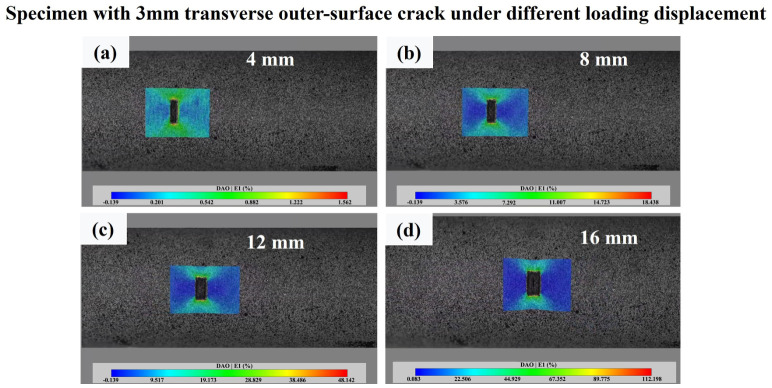
Surface strain distribution of the specimen with 3 mm transverse outer-surface crack at different loading displacements: (**a**) 4 mm; (**b**) 8 mm; (**c**) 12 mm; (**d**) 16 mm.

The 3 mm transverse outer-surface crack specimen in Figure 11 and Table 8 also shows a clear crack-type effect, although its behavior differs from that of the 1 mm case. At 4 mm, distinct high-strain regions have already formed at the two crack tips, indicating that the larger surface crack can perturb the local strain field much more effectively from the early stage of loading. Quantitatively, *ε*_1,*max*_ is approximately 0.42%, *K_ε_* is approximately 2.21, and *G_ε_* is approximately 0.040%/mm, all of which are higher than those of the 1 mm outer-surface crack at the same loading displacement. This shows that increasing the surface-crack length enhances the local notch effect even when the crack does not penetrate the full wall thickness. At 8 mm, these high-strain zones intensify further and develop a more obvious symmetric pattern around the two crack tips, suggesting that plastic strain is beginning to accumulate preferentially at the crack tips. At this displacement, *K_ε_* increases to approximately 3.12, and *G_ε_* increases to approximately 0.82%/mm, indicating a sharper strain transition from the crack-tip region to the surrounding lower-strain region. As the displacement increases to 12 and 16 mm, the crack-tip-dominated feature becomes increasingly evident, and the crack vicinity gradually becomes the dominant local deformation region. This trend is reflected by the progressive increase in *K_ε_* from approximately 3.22 to 3.35 and by the increase in *G_ε_* from approximately 2.60%/mm to 7.50%/mm. Meanwhile, the localization width decreases from approximately 8.6 mm to 7.4 mm, showing that plastic deformation becomes increasingly confined to the crack-tip region. In other words, once the surface crack size reaches 3 mm, it can significantly modulate the strain distribution in the middle gauge region at a relatively early stage and establish a localization tendency centered on the crack tips. Even so, this localization remains more confined, develops more slowly, and is less intense than that observed in the 3 mm through-wall transverse crack specimen. Under comparable loading displacements, the through-wall crack produces stronger strain concentration and earlier signs of instability. For example, the 3 mm outer-surface crack gives *K_ε_* = 3.12 at 8 mm, whereas the corresponding 3 mm through-wall crack gives *K_ε_* = 4.16, indicating stronger strain amplification after complete wall penetration. The 3 mm surface crack clearly deviates from the deformation pattern of the non-cracked specimen, but its control over the overall deformation field is still weaker than that of the through-wall crack.

A combined comparison of Figure 10 and Figure 11 with Figure 8 and Figure 9 indicates that the effect of crack type originates fundamentally from whether wall continuity is broken through the thickness. A through-wall crack interrupts the entire load-bearing path across the wall thickness, forcing load transfer to be redistributed around the two crack ends [14,34]. This promotes stronger stress concentration and faster local plastic-zone development in the crack-tip vicinity. By contrast, because a continuous ligament remains through the wall thickness, an outer-surface crack allows a greater degree of load redistribution and constrains crack opening as well as local plastic expansion. As a result, strain concentration at the crack tips is reduced and the establishment of a localization band is delayed. The quantitative indicators support this mechanism. For the 1 mm crack, the outer-surface specimen maintains *K_ε_* = 1.35–1.47 before final necking, whereas the 1 mm through-wall crack generally shows *K_ε_* > 2.4, demonstrating the stronger localization capability of a fully penetrating defect. For the 3 mm crack, the outer-surface specimen shows a clear increase in localization severity, but its *K_ε_* and *G_ε_* remain lower and its *ω_loc_* remains larger than those of the corresponding through-wall crack. This difference is especially pronounced for the 1 mm crack: the 1 mm outer-surface crack acts mainly as a local perturbation, whereas the 1 mm through-wall crack already becomes a dominant defect controlling localization. When the crack size increases to 3 mm, the effect of the outer-surface crack becomes much stronger, yet it still does not reach the level of control produced by the through-wall crack of the same size. These results demonstrate that crack type affects not only the intensity of local strain concentration, but also the threshold and rate at which a crack evolves from a local disturbance into a dominant feature influencing the overall deformation behavior. Overall, through-wall cracks are more effective than outer-surface cracks in promoting severe localization and therefore exhibit higher failure sensitivity. This behavior is physically reasonable because a surface crack retains a subsurface ligament that delays crack opening and redistributes plastic deformation, whereas a through-wall crack directly disrupts the load-bearing path across the wall thickness and forces deformation to concentrate around the crack tips.

### 3.5. Effect of Crack Orientation on Strain Distribution

**Figure 12:**
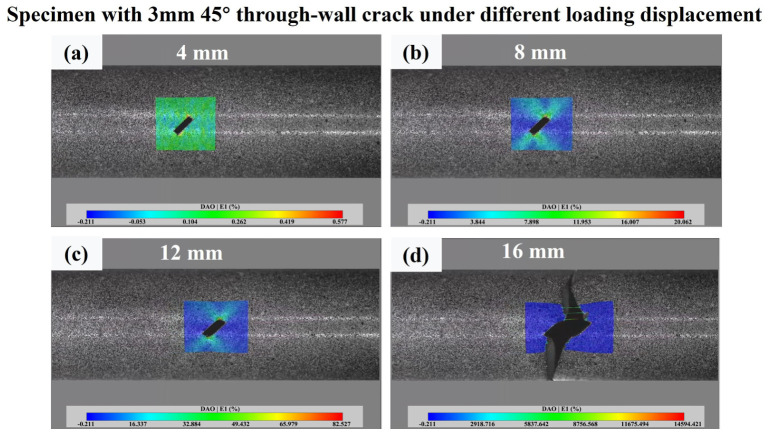
Surface strain distribution of the specimen with 3 mm-45° through-wall crack at different loading displacements: (**a**) 4 mm; (**b**) 8 mm; (**c**) 12 mm; (**d**) 16 mm.

**Figure 13:**
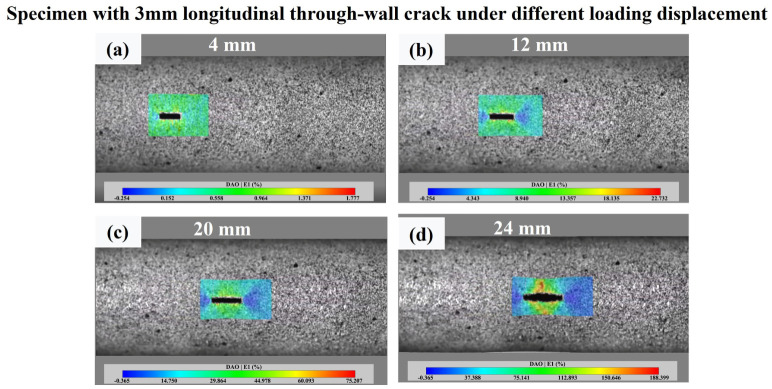
Surface strain distribution of the specimen with a 3 mm-longitudinal through-wall crack at different loading displacements: (**a**) 4 mm; (**b**) 12 mm; (**c**) 20 mm; (**d**) 24 mm.

To evaluate the effect of crack orientation on local strain distribution and its evolution in thin-walled tubular specimens, 3 mm through-wall cracks with different orientations were compared under the same crack size and crack type, as shown in Figure 9 and Figure 12 and Figure 13. Among them, Figure 9 corresponds to the transverse through-wall crack, Figure 12 to the 45° through-wall crack, and Figure 13 to the longitudinal through-wall crack. In contrast to Section 3.3 and Section 3.4, the focus here is no longer on crack size or whether the wall thickness is fully penetrated. Instead, the key issue is how the angle between the crack and the loading direction controls the spatial distribution of the local strain field, the rate at which localization develops, and the sensitivity to instability once wall continuity has already been broken and crack length is held constant. The results show that crack orientation plays a decisive role in localization behavior. It changes not only the intensity of strain concentration, but also the morphology of the high-strain region and the eventual failure mode [25,35].

For the 3 mm-45° through-wall crack specimen, the strain evolution in Figure 12 and Table 9 exhibits the strongest directionality and the earliest signs of instability. At a displacement of 4 mm, noticeable local strain perturbations have already developed near both crack tips, and the high-strain regions begin to align with the inclined crack direction, indicating that the 45° crack strongly affects the surrounding strain field from the early stage of loading. This early disturbance is also reflected by the quantitative DIC indicators: at 4 mm, the effective maximum first principal strain is approximately 0.49%, the strain concentration factor *K_ε_* is approximately 2.72, and the strain-gradient index *G_ε_* is approximately 0.055%/mm. When the displacement increases to 8 mm, strain concentration near the crack tips intensifies rapidly and shows a more pronounced diagonal distribution, suggesting combined normal opening and tangential sliding effects at the two crack tips. At this stage, *K_ε_* increases to approximately 4.51, while *G_ε_* rises to approximately 1.85%/mm, indicating a much sharper strain transition around the inclined crack tips. Local plastic deformation is therefore no longer influenced by a single opening-dominated mode. At 12 mm, this orientation effect becomes even more evident. The high-strain regions continue to expand from the crack tips and gradually develop into a clearer inclined localization pattern, indicating that the strain field in the middle gauge region is already strongly controlled by the crack direction. Quantitatively, the effective maximum first principal strain reaches approximately 76.0%, *K_ε_* further increases to approximately 4.69, and the localization width decreases to about 3.9 mm. These changes show that plastic deformation is rapidly confined to a narrow inclined band near the crack tips. At 16 mm, obvious crack extension and macroscopic fracture have already occurred. This stage should therefore be regarded as a post-instability state. The extremely high apparent strain shown in Figure 12d is mainly associated with crack opening, tangential sliding, displacement discontinuity, and local DIC decorrelation near the fractured region; it should not be interpreted as the true microscopic material strain at the crack tip. This means that the 45° through-wall crack enters the instability and failure stage earlier than the transverse through-wall crack of the same size. In other words, among the three orientations, the 45° crack exhibits the strongest localization capability and the highest failure sensitivity. This behavior contrasts with that of the 3 mm transverse through-wall crack discussed in Section 3.3. The transverse crack also produced strong crack-tip strain concentration, but, at the same displacement, it mainly showed a symmetric tip-controlled localization pattern, whereas the 45° crack evolved more rapidly into an oblique failure mode influenced by tensile-shear coupling. This is physically reasonable because the inclined crack plane experiences both a normal opening component and a tangential shear component under axial tension. The two components act together. As a result, mixed Mode I/Mode II deformation promotes faster inclined localization and earlier macroscopic instability.

By comparison, the strain evolution of the 3 mm longitudinal through-wall crack specimen is much more moderate, as shown in the revised Figure 13 and Table 10. At 4 mm, only weak local perturbations are observed around the crack, and the overall strain field remains relatively smooth, indicating that the longitudinal crack has only a limited influence on local plastic flow during the early loading stage. The corresponding quantitative indicators also remain low: *K_ε_* is approximately 1.88, and *G_ε_* is only about 0.045%/mm. When the displacement increases to 12 mm, a more visible nonuniform strain distribution begins to appear in the crack vicinity, but the high-strain region does not rapidly develop into a strong localization core at the crack ends, as observed for the transverse and 45° cracks. Instead, strain accumulates more gradually and remains relatively localized. At this displacement, *K_ε_* increases only slightly to approximately 2.11, and the localization width remains large, approximately 10.2 mm, indicating that deformation is still distributed over a relatively broad region around the crack. With further loading to 20 mm, strain concentration near the crack becomes more evident, and the high-strain region gradually spreads into the local ligaments adjacent to the crack, indicating that the longitudinal crack is beginning to exert a clearer influence on the strain field in the middle gauge section. This gradual development is reflected by the increase in *K_ε_* to approximately 2.45 and by the increase in *G_ε_* to approximately 3.20%/mm. At 24 mm, a distinct high-strain concentration has formed around the crack region, yet the specimen still does not exhibit the rapid crack extension and macroscopic fracture already observed in the 45° crack specimen. Even at this later stage, *K_ε_* reaches only approximately 2.84, and the localization width remains about 8.4 mm, which is still wider than that of the 45° crack at 12 mm. This indicates that the longitudinal crack can continuously perturb the local strain field and eventually develop a noticeable localization pattern, but the onset of instability is clearly delayed compared with the 45° and transverse cracks. Because the longitudinal crack is nearly parallel to the axial loading direction, the applied load generates only a limited opening effect normal to the crack plane. The crack-driving force at the tips is therefore relatively weak, and local plastic deformation is expressed mainly through gradual strain redistribution in the ligaments surrounding the crack rather than through rapid crack-driven instability. Thus, the longitudinal through-wall crack exhibits a slower localization process, a weaker strain-amplification effect, and a lower failure sensitivity under axial tension.

A combined comparison of Figure 9, Figure 12 and Figure 13 demonstrates that, when crack size and through-wall condition are fixed, crack orientation markedly changes the dominant mechanism of the local strain field. The transverse through-wall crack is characterized mainly by a symmetric opening-type strain concentration at both crack tips, representing a typical crack-tip-dominated localization mode. The 45° through-wall crack, however, is subjected simultaneously to normal opening and tangential shear components, and therefore develops the most pronounced mixed-mode localization pattern and triggers macroscopic failure at the earliest stage. The longitudinal through-wall crack shows the slowest buildup of strain concentration, a relatively weaker localization tendency, and the ability to sustain a larger loading displacement before entering a clearly concentrated deformation stage. This orientation effect is also evident from the DIC-derived indicators. At 8 mm, the 45° crack gives *K_ε_* = 4.51, which is slightly higher than that of the 3 mm transverse through-wall crack, *K_ε_* = 4.16, and much higher than that of the longitudinal crack, even at 12 mm, *K_ε_* = 2.11. Moreover, the localization width of the 45° crack decreases rapidly from approximately 5.8 mm at 4 mm to 3.9 mm at 12 mm, whereas the longitudinal crack maintains a much wider localization zone of approximately 8.4–10.8 mm over the examined displacement range. In this sense, Section 3.3 showed that crack size determines the severity and onset of localization, whereas Section 3.4 demonstrated that crack type influences whether the load-transfer path through the wall thickness is completely interrupted. The present section further shows that crack orientation determines how localization develops and along which path the structure ultimately becomes unstable. Overall, for the 3 mm through-wall crack specimens, the severity can be ranked as 45° crack > transverse crack > longitudinal crack. This ranking is supported not only by the strain-contour morphology, but also by the quantitative DIC indicators, including *ε*_1,*max*_, *K_ε_*, *G_ε_*, *ω_loc_*, and *η_HS_*. This finding indicates that, in structural integrity assessment of defective thin-walled tubular components, considering crack size or crack type alone is not sufficient. Crack orientation is also a key geometrical parameter controlling local failure sensitivity and failure mode.

## 4. Discussion

### 4.1. Comparative Effects of Crack Size, Crack Type, and Crack Orientation

The above results indicate that crack size, crack type, and crack orientation affect strain localization in different but interconnected ways. Crack size primarily controls the intensity and development rate of strain concentration. For transverse through-wall cracks, increasing the crack length from 1 mm to 3 mm increases the normalized crack length from *λ* = 0.05 to *λ* = 0.15. It also reduces the remaining ligament ratio and leads to a higher *ε*_1,*max*_, larger *K_ε_*, steeper *G_ε_*, and smaller *ω_loc_*. These changes suggest that a larger through-wall crack reduces the effective load-bearing section and accelerates the transition from weak crack-tip perturbation to crack-tip-controlled localization.

Crack type mainly determines whether the load-transfer path through the wall thickness is completely interrupted. Through-wall cracks produce stronger strain concentration and earlier localization than outer-surface cracks because the wall-thickness continuity is fully broken. By contrast, outer-surface cracks retain a subsurface ligament. This remaining ligament can still carry part of the applied load and redistribute plastic deformation, thereby delaying the development of severe crack-tip localization. This explains why the 1 mm outer-surface crack behaves mainly as a local perturbation, whereas the 1 mm through-wall crack already shows a clear crack-tip-controlled localization tendency. When the crack length increases to 3 mm, the influence of the outer-surface crack becomes stronger. Even so, it remains weaker than that of the corresponding through-wall crack.

Crack orientation further changes the localization mode and failure path. For the 3 mm through-wall cracks, the 45° crack exhibits the strongest directionality and the earliest macroscopic instability. This is because the inclined crack plane is subjected to both normal opening and tangential shear components under axial tension. The transverse crack mainly produces a symmetric opening-dominated localization pattern at the two crack tips. The longitudinal crack behaves differently. It shows the weakest crack-opening tendency and the slowest localization development. Therefore, the severity of the 3 mm through-wall cracks can be ranked as follows: 45° crack > transverse crack > longitudinal crack.

### 4.2. Physical Mechanism of Strain Localization

The observed localization behavior can be understood from the perspective of local load redistribution and ductile plastic deformation. In the non-cracked specimen, plastic deformation evolves from a relatively uniform state to distributed strain redistribution and finally to central necking. The instability position is therefore influenced mainly by the intrinsic plastic evolution of the material and geometric necking instability. Once a crack is introduced, this deformation process changes. The local load-transfer path is disturbed, the crack tips become preferred sites for strain accumulation, and plastic deformation progressively shifts from a globally distributed mode to a crack-tip-controlled mode.

For through-wall cracks, the entire load path across the wall thickness is interrupted. The applied load must be transferred around the two crack tips, resulting in stronger local stress and strain concentration. For outer-surface cracks, the situation is less severe. The remaining subsurface ligament delays crack opening and allows partial load redistribution, thereby reducing the degree of crack-tip localization. For the 45° inclined crack, the axial tensile load is resolved into both normal and shear components on the crack plane. The normal component promotes crack opening, whereas the shear component promotes crack-face sliding and diagonal plastic flow. This mixed Mode I/Mode II effect explains the inclined localization band and the earlier failure of the 45° crack specimen.

The role of the material should also be considered. X46 steel exhibits a ductile ferrite–pearlite response, and the fracture morphology shows dimples, microvoids, and tearing ridges. Thus, the failure process is not a purely geometric notch effect. Crack geometry determines where strain concentration occurs and how the localization path develops. The material ductility and strain-hardening capacity, however, determine how much plastic deformation can be redistributed before local instability or fracture. The observed failure behavior is therefore controlled by the coupling between crack-induced local load redistribution and the ductile plastic response of the material.

### 4.3. Tensile Properties and Fracture Mechanism of X46 Steel

Previous studies on cracked pipelines and tubular specimens have shown that crack geometry, crack depth, crack location, and constraint condition can strongly influence crack-driving force, *J*-integral, CTOD, fracture toughness, strain capacity, and crack-tip constraint. *J*- and CTOD-based approaches have therefore been widely used in metallic fracture toughness testing and pipeline integrity assessment [36]. For pipeline steels, SE(T)/SENT specimens are commonly adopted to characterize *J*-resistance behavior and to reduce the excessive conservatism associated with high-constraint specimens [37,38]. Surface-cracked pipelines and girth-weld configurations have also been assessed using *J*-integral, CTOD, reference stress, and fully plastic crack-driving-force solutions [39]. More recently, DIC-based methods have been increasingly applied to quantify crack-tip deformation, full-field strain evolution, and even *J*-integral estimation in cracked specimens [40,41].

The present results are consistent with these studies in showing that crack geometry plays a critical role in local deformation and failure sensitivity. The contribution of this work, however, is not merely to reconfirm this general trend. Instead, this study provides a direct DIC-based experimental comparison of full-field strain evolution in X46 thin-walled tubular specimens under the same tensile-dominated loading condition. Crack size, crack type, and crack orientation were controlled separately. This enables their individual effects on strain localization to be distinguished more clearly. In addition, quantitative DIC indicators, including *ε*_1,*max*_, *K_ε_*, *G_ε_*, *ω_loc_*, and *η_HS_*, were introduced to complement the strain-contour observations. These indicators allow the localization process to be compared numerically rather than only visually. As a result, the present work clarifies how different crack geometries reconstruct the local strain field and promote the transition from distributed plastic deformation to crack-tip-controlled localization.

### 4.4. Engineering Implications and Limitations

The present results have practical implications for defect severity ranking and strain-based integrity assessment of thin-walled pipeline components. Larger through-wall cracks and 45° inclined cracks produce stronger strain concentration and earlier localization. Small outer-surface cracks, in contrast, have a weaker influence on the global deformation field. Therefore, crack size, wall-penetration state, and crack orientation should be considered together when prioritizing defects for inspection and assessment. The DIC-derived indicators used in this work can also provide experimental references for validating numerical damage models and strain-based assessment methods.

Several limitations should also be noted. First, all tests were conducted under monotonic quasi-static tensile loading at room temperature. This loading condition was selected as a simplified tensile-dominated representation of the membrane stress state of thin-walled pipelines under constant internal pressure. However, the present tests did not directly include internal pressure or the multiaxial stress state of a pressurized pipe. Second, cyclic loading, environmental degradation, corrosion, hydrogen effects, and temperature effects were not considered. Third, only API 5L X46 steel and a wall thickness of 2 mm were investigated. Finally, the cracks were idealized EDM-prefabricated defects, which are wider and more regular than typical fatigue or corrosion cracks. Therefore, the present results should be regarded as baseline information for understanding crack-geometry-controlled strain localization under monotonic tensile deformation. More realistic service conditions and crack morphologies should be further examined through combined experimental and numerical approaches.

## 5. Conclusions

Based on uniaxial tensile tests and DIC-based full-field strain measurements, the local deformation and failure behavior of X46 pipeline steel thin-walled tubular specimens without cracks and with different crack geometries were systematically investigated. The main conclusions are as follows:

1. X46 pipeline steel exhibited a favorable strength-ductility balance at room temperature, as reflected by an average yield strength of 324.0 MPa, an ultimate tensile strength of 553.5 MPa, and an elongation of 27.0%. The fracture surface was characterized by numerous dimples, microvoids, and tearing ridges, with a small number of spherical inclusions observed inside some dimples, indicating a typical ductile fracture mechanism influenced by microvoid nucleation, growth, and coalescence.
2. The non-cracked specimen exhibited a clear stage-wise evolution from uniform deformation to strain redistribution and then to enhanced localization, with the final high-strain zone consistently developing in the middle of the gauge section. This indicates that, in the absence of crack-induced disturbance, the onset of instability was influenced mainly by the intrinsic plastic deformation of the material and geometric instability associated with necking.
3. Once a crack was introduced, localization still developed within the middle gauge region, but its dominant form changed from a continuous, centrally evolving strain concentration pattern to a crack-tip-controlled mode. Increasing crack size markedly intensified strain concentration in the crack vicinity and accelerated the onset of localization. In particular, the 3 mm through-wall transverse crack specimen exhibited earlier crack-tip-dominated deformation and earlier signs of local instability than the 1 mm through-wall transverse crack specimen.
4. Crack type determined whether the continuous load-bearing path through the wall thickness was interrupted. Through-wall cracks generated much stronger crack-tip strain concentration and established localization bands more rapidly than outer-surface cracks. The 1 mm outer-surface crack acted mainly as a local perturbation, and failure was still influenced primarily by necking in the middle of the gauge section, whereas the through-wall crack of the same size already exerted clear control over localization evolution. Even at 3 mm, the outer-surface crack remained less dominant than the corresponding through-wall crack.
5. When crack size and crack type were fixed, crack orientation further determined the localization mode and instability path. The 3 mm-45° through-wall crack exhibited the strongest tensile-shear-coupled localization and the earliest macroscopic instability; the 3 mm transverse through-wall crack was characterized mainly by symmetric opening-type strain concentration; and the 3 mm longitudinal through-wall crack showed the slowest localization development and the weakest localization tendency. Accordingly, the relative severity can be ranked as follows: 45°crack > transverse crack > longitudinal crack.

Overall, crack geometry influences strain distribution, localization development, and final failure behavior in X46 pipeline steel thin-walled tubular specimens through its effects on local load transfer and crack-tip plastic evolution. In essence, crack size influences the intensity of localization, crack type controls the continuity of the load path, and crack orientation determines the mode of instability. These findings provide a direct experimental basis for the structural integrity assessment and failure prediction of defective oil and gas pipelines.

## Figures and Tables

**Table 1:** Chemical composition of X46 steel (wt.%).

Materials	C	Mn	Si	S	P	Nb	Ti	Al
X46	0.05	1.13	0.15	0.004	0.013	0.018	0.021	0.013

**Table 2:** Crack geometry settings.

Specimen Type	Specimen Specifications	Crack Length	Crack Type
Hollow round tube	Outer diameter 20 mm Inner diameter 16 mm	0 mm	No crack
		1 mm	0° outer surface crack
		1 mm	0° through-wall crack
		3 mm	0° outer surface crack
		3 mm	0° through-wall crack
		3 mm	45° through-wall crack
		3 mm	90° through-wall crack

**Table 3:** X46 steel room temperature tensile properties.

Specimen Number	Yield Strength/MPa	Tensile Strength/MPa	Elongation/%
X46-1	308.3	543.5	27.6
X46-2	325.2	554.3	26.3
X46-3	338.5	562.7	27.1

**Table 4:** Quantitative DIC indicators of the non-cracked specimen at different loading displacements.

Loading Displacement	*ε*_1,*max*_/%	*ε*_1,*far*_/%	*K_ε_*/%·mm^−1^	*G_ε_*/%·mm^−1^	*ω_loc_*/mm	*η_HS_*/%
4 mm	0.715	0.436	1.64	0.0026	55.1	5.18
12 mm	9.884	7.523	1.31	0.061	56.3	19.74
20 mm	20.345	16.145	1.26	0.128	52.4	50.26
28 mm	74.053	25.557	2.90	2.89	26.8	23.46

**Table 5:** Quantitative DIC indicators of the specimen with a 1 mm transverse through-wall crack.

Loading Displacement	*ε*_1,*max*_/%	*ε*_1,*far*_/%	*K_ε_*/%·mm^−1^	*G_ε_*/%·mm^−1^	*ω_loc_*/mm	*η_HS_*/%
4 mm	0.34	0.12	2.83	0.04	5.8	3.8
8 mm	8.7	3.6	2.42	0.72	6.4	5.6
16 mm	74.5	28.6	2.60	5.7	7.2	6.3
20 mm	650.0	184.9	3.52	38.8	5.0	4.9

**Table 6:** Quantitative DIC indicators of the specimen with a 3 mm transverse through-wall crack.

Loading Displacement	*ε*_1,*max*_/%	*ε*_1,*far*_/%	*K_ε_*/%·mm^−1^	*G_ε_*/%·mm^−1^	*ω_loc_*/mm	*η_HS_*/%
4 mm	0.55	0.19	2.89	0.06	6.0	5.2
8 mm	16.1	3.87	4.16	1.53	5.5	7.4
12 mm	58.8	14.5	4.06	5.54	4.8	8.1
16 mm	216.8	53.8	4.03	20.4	4.5	6.7

**Table 7:** Quantitative DIC indicators of the specimen with a 1 mm transverse outer-surface crack.

Loading Displacement	*ε*_1,*max*_/%	*ε*_1,*far*_/%	*K_ε_*/%·mm^−1^	*G_ε_*/%·mm^−1^	*ω_loc_*/mm	*η_HS_*/%
4 mm	0.22	0.15	1.47	0.015	34.5	2.6
12 mm	6.5	4.7	1.38	0.18	38.0	5.8
20 mm	18.6	13.8	1.35	0.42	41.5	9.6
30 mm	82.0	34.5	2.38	2.15	28.5	18.4

**Table 8:** Quantitative DIC indicators of the specimen with a 3 mm transverse outer-surface crack.

Loading Displacement	*ε*_1,*max*_/%	*ε*_1,*far*_/%	*K_ε_*/%·mm^−1^	*G_ε_*/%·mm^−1^	*ω_loc_*/mm	*η_HS_*/%
4 mm	0.42	0.19	2.21	0.04	12.50	4.80
8 mm	10.6	3.40	3.12	0.82	10.20	7.50
12 mm	32.5	10.1	3.22	2.60	8.60	9.40
16 mm	85.0	25.4	3.35	7.50	7.40	10.80

**Table 9:** Quantitative DIC indicators of the specimen with a 3 mm-45° through-wall crack.

Loading Displacement	*ε*_1,*max*_/%	*ε*_1,*far*_/%	*K_ε_*/%·mm^−1^	*G_ε_*/%·mm^−1^	*ω_loc_*/mm	*η_HS_*/%
4 mm	0.49	0.18	2.72	0.055	5.80	5.60
8 mm	18.50	4.10	4.51	1.85	4.90	8.30
12 mm	76.00	16.20	4.69	7.40	3.90	9.50
16 mm	Post-instability	-	-	-	-	-

**Table 10:** Quantitative DIC indicators of the specimen with a 3 mm longitudinal through-wall crack.

Loading Displacement	*ε*_1,*max*_/%	*ε*_1,*far*_/%	*K_ε_*/%·mm^−1^	*G_ε_*/%·mm^−1^	*ω_loc_*/mm	*η_HS_*/%
4 mm	1.35	0.72	1.88	0.045	10.80	4.50
12 mm	16.00	7.60	2.11	0.75	10.20	6.20
20 mm	60.00	24.50	2.45	3.20	9.10	8.00
24 mm	145.00	51.00	2.84	7.10	8.40	9.80

## Data Availability

The original contributions presented in this study are included in the article. Further inquiries can be directed to the corresponding author.
